# Myocardial Work Indices Predict Survival Post TAVI in Aortic Stenosis Patients

**DOI:** 10.3390/jcm15041645

**Published:** 2026-02-22

**Authors:** Michał Jakub Błaszkiewicz, Tomasz Grzegorz Witkowski, Wojciech Bombała, Michał Kosowski, Piotr Kübler, Krzysztof Aleksandrowicz, Ewa Anita Jankowska, Marcin Protasiewicz

**Affiliations:** 1 Institute of Heart Diseases, Jan Mikulicz-Radecki University Hospital, Borowska 213, 50-556 Wroclaw, Poland; 2 Institute of Heart Diseases, Wroclaw Medical University, 50-367 Wroclaw, Poland; 3 Statistical Analysis Centre, Wroclaw Medical University, 50-367 Wroclaw, Poland; 4 Department of Physiotherapy, Faculty of Health Sciences, Wroclaw Medical University, 50-367 Wroclaw, Poland

**Keywords:** aortic stenosis, transcatheter aortic valve implantation, myocardial work, left ventricle systolic function, mortality

## Abstract

**Background**: Left Ventricle Myocardial Work (LVMW) has shown utility in assessing patients with aortic stenosis (AS) in recent studies. In the present study, we evaluated the predictive value and optimal cut-off values of LVMW parameters measured prior to TAVI that may be associated with increased mortality in AS patients. **Methods**: A total of 116 consecutive patients who were qualified for TAVI between March 2021 and November 2022 were evaluated. Pre-procedural LVMW indices (GWI, GCW, GWW, and GWE) were assessed and long-term survival was analysed. Survival and influencing factors were evaluated using univariate and multivariate Cox proportional hazard models, with significant factors subsequently included in cut-off analysis. **Results**: The median survival time following the TAVI procedure was 1404 (1143–1549) days, with a maximum observation period of 1721 days. All-cause mortality during the follow-up period reached 29%. Multivariate analysis revealed that EF, GLS, GWI, GWE and GCW before TAVI were independent predictors of all-cause mortality. We identified 1975 mmHg, 1497 mmHg and 85% as optimal cut-off values for GCW, GWI and GWE, which allow for significant stratification of patients according to risk. **Conclusions**: In this analysis, baseline-assessed parameters such as GLS, GWI, GWE, and GCW emerged as independent predictors of all-cause mortality. The proposed cut-off values clearly separated patient groups with different survival outcomes.

## 1. Introduction

Aortic stenosis (AS), characterised by aortic valve calcification and reduced valve area (AVA), causes left ventricular outflow tract (LVOT) obstruction, followed by increased afterload. To maintain blood ejection through the stenotic valve, left ventricular systolic pressure (LVSP) must increase. Over time, sustained pressure overload leads to myocardial damage and impaired left ventricle (LV) function.

Although, at an early stage of the disease, left ventricle ejection fraction (LVEF) may be preserved, the Global Longitudinal Strain (GLS), a well-known marker of subclinical LV deterioration, often declines earlier [1,2,3]. However, LVEF and GLS, commonly used for LV function, are load-dependent and may misrepresent true performance by overlooking LVSP changes caused by AS. Although TAVI provides uncontested clinical benefits for AS patients, a significant proportion of them still experience high mortality in the medium and long term after the procedure [4,5]. This prompts the question of whether conventionally used LV parameters like LVEF and GLS are sufficient for early detection of LV functional deterioration and reliable prediction of TAVI outcomes. Left ventricular myocardial work (LVMW) is an emerging echocardiographic technique for evaluating myocardial function designed to address the limitations of LVEF and GLS. It enables early detection of LV dysfunction, whether it is caused by myocardial injury or alterations in load conditions [6]. LVMW can be effectively used to screen a broader range of patients, including those with cardiomyopathies, hypertension, heart failure, and AS [7,8,9,10]. Determination of individual LVMW parameter values requires measurement of LVSP. To ensure accurate assessment, the echocardiographic method used for this measurement must account for both the mean aortic valve gradient and the aortic systolic pressure [10]. Recent studies have demonstrated that in patients with severe AS, LVSP can be accurately estimated noninvasively by adding the Doppler-derived mean aortic gradient to the brachial cuff Systolic Blood Pressure [11]. Ribic et al. further validated the reliability of noninvasive LVSP assessment in the AS population [12]. Changes in LVMW parameters are well-recognised as significant predictors of long-term outcomes and may serve as valuable indicators for assessing risk in patients undergoing TAVI [13,14,15]. This raises the question of whether there are well-defined cut-off values for individual baseline LVMW parameters that can identify groups of patients with aortic stenosis at increased risk of mortality. To address this question, we analysed LVMW parameters in consecutive patients undergoing TAVI and evaluated their long-term survival.

## 2. Materials and Methods

### 2.1. Study Design

We conducted a retrospective analysis of 116 consecutive patients with severe aortic stenosis who were qualified for the TAVI procedure by a multidisciplinary Heart Team between March 2021 and November 2022. Informed consent for the procedure was obtained from all participants. This study was approved by the appropriate institutional bioethics committee (KB 792/2020) and was conducted in accordance with the Declaration of Helsinki (1975), as confirmed by prior approval from the institutional review board.

### 2.2. Blood Pressure Measurements

Blood pressure (BP) was measured in all patients immediately prior to each echocardiographic examination, using a noninvasive cuff on the left arm, in accordance with current clinical guidelines [16].

### 2.3. Echocardiographic Examination

Transthoracic echocardiography (TTE) was performed using the Vivid S70 ultrasound system (GE Healthcare, Chicago, IL, USA) one day prior to the TAVI procedure, as part of standard clinical care. To evaluate AS severity, the following parameters were assessed: LVOT diameter, pulsed-wave Doppler (PW) flow, aortic jet velocity via continuous-wave Doppler (CW), and aortic valve area (AVA) calculated using the Bernoulli equation. For assessment of LVEF, GLS, and LVMW, apical 2-chamber, 3-chamber, and 4-chamber views were acquired. All images were analysed using EchoPAC software (version 203, GE Healthcare, Chicago, IL, USA) in accordance with current echocardiographic guidelines. LVEF was determined using the biplane Simpson’s method, while GLS was calculated as the average peak longitudinal strain across all apical views.

### 2.4. Myocardial Work Evaluation

LVMW indices were obtained by first selecting optimal apical views for each echocardiographic study, followed by an analysis of GLS. Brachial blood pressure measurements were manually incorporated into the EchoPAC software and synchronised with GLS data to generate noninvasive pressure–strain loops (PSLs). The systolic cuff pressure served as an estimate for LVSP. The timing of cardiac valve events was identified from the apical 3-chamber view. Based on these data, the following myocardial work parameters were calculated:

- Global Work Index (GWI, mm Hg%): Represents the total area within the pressure–strain loop, measured from mitral valve closure to opening. It reflects the overall myocardial work performed during both systole and diastole, offering a comprehensive assessment of left ventricular function.
- Global Constructive Work (GCW, mm Hg%): Reflects the effective myocardial work of the left ventricle, defined by myocardial fibre shortening during systole and lengthening during diastole.
- Global Wasted Work (GWW, mm Hg%): Quantifies inefficient myocardial activity, indicating left ventricular dyssynchrony. It is defined by myocardial fibre lengthening during systole and shortening during isovolumic relaxation—mechanical actions that do not contribute to effective cardiac output.
- Global Work Efficiency (GWE, %): Reflects the proportion of effective myocardial work relative to the total work performed during both systole and diastole. It is calculated using the formula: (GCW/[GCW + GWW]) × 100%.

### 2.5. Statistical Analysis

Statistical analyses were performed using Statistica 13.0 (StatSoft Inc., Tulsa, OK, USA) and R. Both the Kolmogorov–Smirnov one-sample test and the Shapiro–Wilk test were employed to assess distribution. The continuous variables are presented as mean ± standard deviation or as median and interquartile range (Q1–Q3) as appropriate, while the categorical variables are displayed as numbers and percentages.

We analysed the survival times and influencing factors with univariate and multivariate Cox proportional hazard regression models. Notably, the results of the univariate model were not used as the basis for selecting variables for inclusion in the multivariate model. The assumptions underlying the Cox regression models were evaluated using the Schoenfeld residuals test. To determine the cut-off points of the LVMWi, we used functions surv_cutpoint() and surv_categorize() from package ‘maxstat’ 2 (R version 4.5.0, https://www.R-project.org/ (accessed on 25 November 2025); RStudio version 2025.05.0). These functions identify the optimal cutpoint for one or multiple continuous variables at once, using the maximally selected rank statistics from ‘maxstat’ in the R package. This is an outcome-oriented method providing a value of a cutpoint that corresponds to the most significant relation with the outcome (here, survival). For all statistical tests, a *p*-value > 0.05 was deemed significant.

## 3. Results

**Figure 1:**
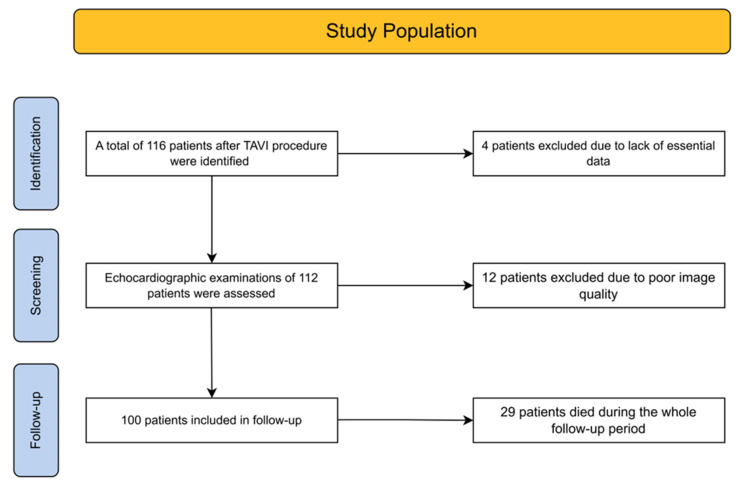
A flowchart of the study population. TAVI—Transcatheter Aortic Valve Implantation.

Sixteen patients were excluded from the analysis due to incomplete data or suboptimal echocardiographic image quality. Ultimately, 100 patients were included in the final cohort, with a mean age of 78 ± 7.1 years. A flowchart illustrating the study population is shown in Figure 1.

The median survival time following the TAVI procedure was 1404 (1143–1549) days, with a maximum observation period of 1721 days. All-cause mortality during the follow-up period reached 29%. Comprehensive clinical and laboratory baseline characteristics are summarised in Table 1, while baseline echocardiographic parameters are detailed in Table 2.

The univariate model presenting the analysed predictors of all-cause mortality is shown in Table 3.

To assess the impact and independence of the factors, we developed four distinct multivariate Cox proportional hazard models, using different sets of variables and their interactions:

Model I = LVMWi + DM + CKD + AF + cID + LVMW*DM + LVMW*CKD + LVMW*AF + LVMW*ID

Model II = LVMWi + DM + CKD + MI + cID + LVMW*DM + LVMW*CKD + LVMW*MI + LVMW*ID

Model III = LVMWi + DM + CKD + AF + TSAT < 20% + LVMW*DM + LVMW*CKD +LVMW*AF + LVMW*TSAT < 20%

Model IV = LVMWi + DM + CKD + MI + TSAT < 20% + LVMW*DM + LVMW*CKD +LVMW*MI + LVMW*TSAT < 20%

where:

LVMWi—Selected Left Ventricle Myocardial Work index before the procedure. DM—Diabetes Mellitus. CKD—Chronic Kidney Disease. AF—Atrial Fibrillation. MI—Myocardial Infarction. cID—Iron deficiency (the iron-deficiency group of patients was defined by the classic definition of ferritin level < 100 µg/L or serum ferritin 100–299 µg/L in combination with TSAT < 20%). *—interaction.

TSAT < 20%—iron deficiency, defined by the novel definition of TSAT < 20%.

Multivariate analysis revealed that EF, GLS, GWI, GWE, and GCW before TAVI were independent predictors of all-cause mortality. Other analysed parameters, including AF, CKD, MI, and both types of iron deficiency, were either not statistically significant or did not meet the assumptions of the model. A complete summary of multivariate models is presented in Table 4.

**Figure 2:**
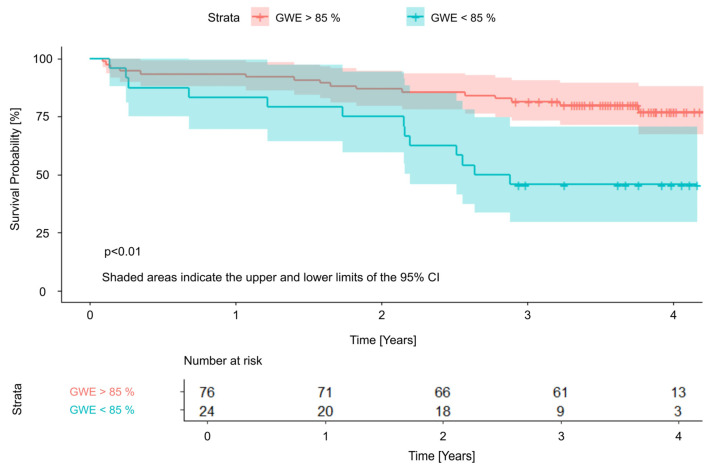
Survival curves and cut-off value for Global Work Efficiency. GWE—Global Work Efficiency; CI—Confidence Interval.

**Figure 3:**
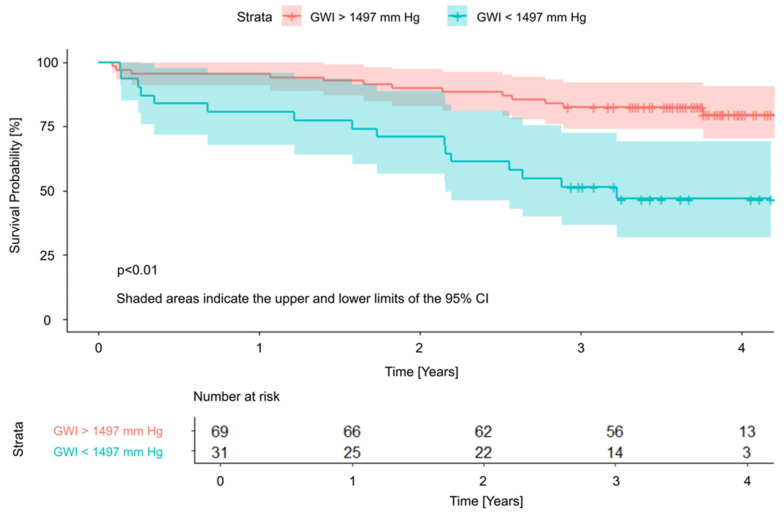
Survival curves and cut-off value for Global Work Index. GWI—Global Work Index; CI—Confidence Interval.

**Figure 4:**
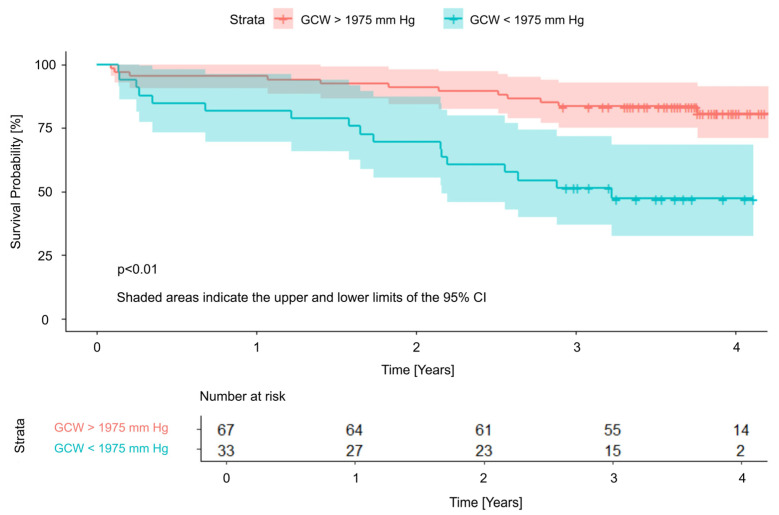
Survival curves and cut-off value for Global Constructive Work. GCW—Global Constructive Work; CI—Confidence Interval.

The independent LVMWi predictors were subsequently included in a cut-off point analysis, in which all parameters demonstrated statistically significant differences between the two groups divided according to the determined cut-off values. Significant threshold values were 1975 mmHg, 1497 mmHg, and 85% for GCW, GWI, and GWE, respectively. Survival curves with cut-off values are presented in Figure 2, Figure 3 and Figure 4.

## 4. Discussion

In this study, we aimed to evaluate the predictive value and determine optimal cut-off points of LVMW indices in patients with AS undergoing TAVI. Our findings showed that mortality within the analysed population remained notable, highlighting the need for reliable tools that enable early identification of patients requiring intervention and support effective risk stratification. We believe that analysis of LVMW indices may, as demonstrated in previous studies of stress echocardiography, improve risk stratification in patients with aortic stenosis [17] and facilitate identification of early myocardial dysfunction [18].

While it is well established that LVMW parameters change significantly following TAVI and may serve as a prognostic factor, these changes vary across patient subgroups [13,15,16]. Although LVMW generally decreases in AS patients after TAVI [11,15,19,20], significantly reduced values measured both pre- and post procedure are linked to clinical symptoms and elevated risk of mortality [10]. This finding may be explained by the differing patterns of LVMW changes between patients with reduced and preserved ejection fraction. Pedersen et al. demonstrated that in patients with reduced left ventricular ejection fraction (LVEF), LVMW indices tend to improve after TAVI. In contrast, those with preserved LVEF may show a post-procedural decline in LVMW, likely reflecting preserved contractile function and a reduction in afterload. Moreover, patients with reduced LVEF consistently display lower LVMW values both before and after the procedure [15]. This suggests a progressive decrease in LVMW as the disease and pressure overload progress. At a certain point, ongoing myocardial stress may lead to irreversible LV remodelling and damage, marked by significant loss of contractility and a pronounced reduction in LVMW. Such a decline may reflect a diminished potential for recovery, ultimately leading to poor clinical outcomes.

In alignment with the literature, our results indicate that LVMW overcomes the major limitation of traditional LV deformation parameters, as they depend on loading conditions. It can be effectively applied to a wide range of patients including those with AS, where the underlying mechanism of the disease is an alteration of normal LV hemodynamic load [7,8,9,21,22]. However, to the best of our knowledge, there are no validated survival cut-off thresholds for LVMW in the AS patients that have been widely validated or confirmed in larger clinical cohorts. In our analysis, patients stratified according to the proposed thresholds showed significant differences in survival curves. Wu et al. [13] reported a very similar mortality rate (23%) in their study group over a comparable observation period and, importantly, also identified the GWI cut-off point associated with survival. Our findings are also consistent with the analyses of Anwer et al. [23], who identified a similar cut-off point, and with Pedersen et al. [15], who demonstrated that each 100 mmHg% increase in LV GWI prior to TAVI was associated with a 4% reduction in the risk of all-cause mortality. All these results, both from other authors’ studies and from our analysis, are the first suggestions indicating that specific LVMW parameters and their exact values should be considered potentially useful in assessing the prognosis of patients undergoing TAVI.

Notably, in our observation, a relatively large proportion of patients (24–33%, depending on the LVMW parameter assessed—Figure 2) fell below the identified cut-off values of higher risk of poor prognosis, indicating that a substantial subgroup qualified for TAVI may have been referred at a too advanced stage of myocardial damage, thereby facing a significantly reduced likelihood of benefiting from the procedure. Implementing specific thresholds for LVMW may assist in determining the optimal timing for aortic valve replacement, helping to intervene before irreversible structural and functional myocardial changes occur, which could otherwise compromise long-term outcomes [24].

Our results lay the foundation for a more in-depth analysis of LVMW alterations throughout the progression of AS, as well as future studies aimed at defining optimal reference ranges for asymptomatic AS patients. Incorporating LVMW assessment into routine AS screening and the TAVI qualification process may support better timing of valve interventions, ultimately enhancing post-procedural cardiac performance and improving prognosis in patients undergoing TAVI.

## 5. Conclusions

Low LVMW values measured before the TAVI procedure are associated with a higher risk of mortality in AS patients. In this analysis, LVEF, GLS, GWI, GWE and GCW assessed at baseline emerged as independent predictors of all-cause mortality. The proposed cut-off values clearly separated patient groups with different survival outcomes. Further studies are needed to clarify the progression of LVMW changes and their value in AS screening and prognosis following TAVI. Validation studies of the cut-off values we propose for individual LVMW parameters may be of particular importance.

## 6. Limitations

Being a single-centre study, our research faces several notable limitations. Primarily, the sample size was limited, with an even smaller subset of survivors. While blood pressure was recorded just before the echocardiographic assessment, potential variations during the examination itself may have impacted the results. Furthermore, underlying comorbidities that may additionally impact cardiac function could have influenced GLS measurements, potentially leading to inaccurate interpretations in some patients. Since our analysis focused on all-cause mortality, we did not specifically account for other comorbidities or factors influencing mortality in the study population beyond the primary valvular disease.

## Figures and Tables

**Table 1:** Clinical and laboratory baseline characteristics.

Parameter	All Patients (100)
Age [years], mean ± SD	78.4 ± 7.1
Male Gender, n (%)	39 (39)
BMI, median (Q1–Q3)	27.52 (24.77–30.96)
6MWT [m], median (Q1–Q3)	277.5 (180–360)
NYHA, median (Q1–Q3)	2 (2–3)
STS Score, mean ± SD	4.1 ± 4.7
Hypertension, n (%)	89 (89)
COPD, n (%)	7 (7)
Pacemaker, n (%)	8 (8)
Prior PCI, n (%)	37 (37)
Prior CABG, n (%)	13 (13)
SBP [mmHg], median (Q1–Q3)	136 (120–148)
DBP [mmHg], median (Q1–Q3)	79 (70–80)
Hyperlipidaemia, n (%)	68 (68)
Peripheral Artery Disease, n (%)	30 (30)
Atrial Fibrillation, n (%)	31 (31)
Previous MI, n (%)	46 (46)
Prior RBBB, n (%)	6 (6)
Prior LBBB, n (%)	7 (7)
Chronic HF, n (%)	35 (35)
Diabetes Mellitus, n (%)	40 (40)
Chronic Kidney Disease, n (%)	40 (40)
eGFR [mL/min/1.73 m^2^], median (Q1–Q3)	60.5 (47–71)
NT-proBNP [pg/mL], median (Q1–Q3)	3030.55 (1006.2–8135.85)
Creatinine [mg/dL], median (Q1–Q3)	1.08 (0.93–1.37)
hsCRP [mg/L], median (Q1–Q3)	3.44 (0.87–11.9)
Hb [g/dL], median (Q1–Q3)	12.5 (11.5–13.3)

6MWT—Six-Minute Walk Test; BMI—Body Mass Index; CABG—Coronary Artery Bypass Graft; COPD—Chronic Obstructive Pulmonary Disease; DBP—Diastolic Blood Pressure; eGFR—Estimated Glomerular Filtration Rate; Hb—Haemoglobin; HF—Heart Failure; hsCRP—High-Sensitivity C-Reactive Protein; LBBB—Left Bundle Branch Block; MI—Myocardial Infarction; NT-proBNP—N-Terminal pro-B-Type Natriuretic Peptide; NYHA—New York Heart Association (functional class); PCI—Percutaneous Coronary Intervention; RBBB—Right Bundle Branch Block; SBP—Systolic Blood Pressure; STS score—Society of Thoracic Surgeons risk score.

**Table 2:** Baseline echocardiographic characteristics.

Parameter	All Patients (100)
AVAi [cm^2^/m^2^], median (Q1–Q3)	0.37 (0.33–0.43)
Vmax [m/s], median (Q1–Q3)	4.39 (4.11–4.81)
Pmean [mmHg], median (Q1–Q3)	47.84 (40.99–59)
EF [%], median (Q1–Q3)	60 (54–64.5)
GLS [%], median (Q1–Q3)	14.4 (17.9–10.35)
GWE [%], median (Q1–Q3)	91 (86–95)
GWI [mmHg·%], median (Q1–Q3)	2198 (1368.5–2779)
GCW [mmHg·%], median (Q1–Q3)	2500.5 (1685.5–3096.5)
GWW [mmHg·%], median (Q1–Q3)	173 (122.5–261)

AVAi—Aortic Valve Area indexed; EF—Ejection Fraction; GCW—Global Constructive Work; GLS—Global Longitudinal Strain; GWE—Global Work Efficiency; GWI—Global Work Index; GWW—Global Wasted Work; Pmean—Mean Transvalvular Pressure Gradient; Vmax—Maximum Aortic Jet Velocity.

**Table 3:** The univariate model presenting the analysed predictors of all-cause mortality.

Parameter	HR (95% CI for HR)	*p*-Value
Male Gender	1.308 (0.6291–2.721)	0.47
Age	1.042 (0.9851–1.102)	0.15
BMI	0.9844 (0.9156–1.058)	0.67
BSA	2.356 (0.3826–14.5)	0.36
Hypertension	0.94 (0.1271–6.951)	0.95
COPD	0.4747 (0.06448–3.494)	0.46
Hyperlipidaemia	1.686 (0.5803–4.898)	0.34
Peripheral Artery Disease	1.854 (0.8753–3.928)	0.11
Atrial Fibrillation	2.126 (0.9844–4.593)	0.055
Pacemaker	2.098 (0.7225–6.092)	0.17
MI	1.607 (0.7726–3.341)	0.2
Prior PCI	1.823 (0.8422–3.945)	0.13
Prior CABG	0.7304 (0.219–2.436)	0.61
Prior LBBB	1.833 (0.5499–6.112)	0.32
CHF	1.465 (0.6927–3.099)	0.32
DM	1.718 (0.8172–3.611)	0.15
Chronic Kidney Disease	1.417 (0.948–2.12)	0.089
GFR	0.9799 (0.9593–1.001)	0.062
NTproBNP	1 (0.9999–1)	0.42
Creatinine	0.7748 (0.4765–1.26)	0.3
hsCRP	0.9939 (0.9718–1.017)	0.6
Hb	0.7742 (0.6352–0.9436)	0.011
TSAT	0.9611 (0.9223–1.001)	0.058
Fe	0.9843 (0.9716–0.9972)	0.017
EF	0.9661 (0.9367–0.9963)	0.028
GLS	0.879 (0.8113–0.9524)	0.002
GWE	0.9404 (0.9032–0.9792)	0.003
GWI	0.9994 (0.9989–0.9998)	0.003
GCW	0.9994 (0.999–0.9998)	0.005
GWW	1.003 (0.9999–1.007)	0.056

BMI—Body Mass Index; BSA—Body Surface Area; CABG—Coronary Artery Bypass Graft; CHF—Chronic Heart Failure; COPD—Chronic Obstructive Pulmonary Disease; DM—Diabetes Mellitus; EF—Ejection Fraction; Fe—Serum Iron; GCW—Global Constructive Work; GFR—Glomerular Filtration Rate; GLS—Global Longitudinal Strain; GWE—Global Work Efficiency; GWI—Global Work Index; GWW—Global Wasted Work; Hb—Haemoglobin; hsCRP—High-Sensitivity C-Reactive Protein; LBBB—Left Bundle Branch Block; MI—Myocardial Infarction; NTproBNP—N-Terminal pro-B-Type Natriuretic Peptide; PCI—Percutaneous Coronary Intervention; Tsat—Transferrin Saturation.

**Table 4:** The complete multivariate model presentation of the analysed predictors of all-cause mortality.

Left Ventricle Myocardial Work Parameters Before TAVI—HR (95% PU); *p*-Value				
Parameter	Model I	Model II	Model III	Model IV
EF	0.9511 (0.9092–0.995); *p* = 0.029	0.975 (0.926–1.0265); *p* = 0.335	0.9492 (0.9044–0.9961); *p* = 0.034	0.9771 (0.9278–1.029); *p* = 0.38
GLS	1.1887 (1.0674–1.3239); *p* = 0.002	1.0896 (0.9657–1.2294); *p* = 0.164	1.2131 (1.0734–1.371); *p* = 0.002	1.1269 (0.9941–1.2775); *p* = 0.062
GWE	0.8897 (0.8238–0.9609); *p* = 0.003	0.9142 (0.843–0.9914); *p* = 0.03	0.8975 (0.8368–0.9625); *p* = 0.002	0.9424 (0.8841–1.0045); *p* = 0.068
GWI	0.999 (0.9984–0.9997); *p* = 0.002	0.9994 (0.9988–0.9999); *p* = 0.018	0.999 (0.9984–0.9997); *p* = 0.003	0.9992 (0.9987–0.9998); *p* = 0.013
GCW	0.9991 (0.9985–0.9996); *p* = 0.002	0.9994 (0.9989–0.9999); *p* = 0.022	0.999 (0.9984–0.9996); *p* = 0.001	0.9993 (0.9988–0.9998); *p* = 0.01
GWW	1.0007 (0.996–1.0054); *p* = 0.786	1.0023 (0.9977–1.0069); *p* = 0.322	1.0019 (0.9966–1.0072); *p* = 0.478	1.0028 (0.998–1.0076); *p* = 0.246

Model I = LVMWi + DM + CKD + AF + cID + LVMW*DM + LVMW*CKD +LVMW*AF +LVMW*ID; Model II = LVMWi + DM + CKD + MI + cID + LVMW*DM + LVMW*CKD +LVMW*MI +LVMW*ID; Model III = LVMWi + DM + CKD + AF + TSAT < 20% + LVMW*DM + LVMW*CKD + LVMW*AF + LVMW*TSAT < 20%; Model IV = LVMWi + DM + CKD + MI + TSAT < 20% + LVMW*DM + LVMW*CKD +LVMW*MI + LVMW*TSAT < 20%; AF—Atrial Fibrillation; CKD—Chronic Kidney Disease; DM—Diabetes Mellitus; EF—Ejection Fraction; GCW—Global Constructive Work; GLS—Global Longitudinal Strain; GWE—Global Work Efficiency; GWI—Global Work Index; GWW—Global Wasted Work; ID—Iron deficiency (iron deficiency group of patients was defined as a ferritin level <100 µg/L or serum ferritin 100–299 µg/L in combination with a TSAT < 20%); LVMWi—Left Ventricle Myocardial Work index (before procedure); MI—Myocardial Infarction; TSAT < 20%—Iron deficiency, defined by the novel definition as TSAT < 20%, *—interaction.

## Data Availability

The original contributions presented in this study are included in the article. Further inquiries can be directed to the corresponding author.

## Abbreviations

The following abbreviations are used in this manuscript:

AS	Aortic Stenosis
AVA	Aortic Valve Area
CI	Confidence Interval
GCW	Global Constructive Work
GLS	Global Longitudinal Strain
GWE	Global Work Efficiency
GWI	Global Work Index
GWW	Global Wasted Work
HR	Hazard Ratio
LV	Left Ventricle
LVEF	Left Ventricular Ejection Fraction
LVMW	Left Ventricular Myocardial Work
LVMWi	Left Ventricular Myocardial Work Index
LVOT	Left Ventricular Outflow Tract
LVSP	Left Ventricular Systolic Pressure
TAVI	Transcatheter Aortic Valve Implantation
TTE	Transthoracic Echocardiography
